# Spatio-temporal analysis of bacillary dysentery in Sichuan province, China, 2011–2019

**DOI:** 10.1186/s12879-021-06738-9

**Published:** 2021-10-03

**Authors:** Yao Zhang, Mengyuan Zhang, Dianju Kang, Wei Sun, Changhong Yang, Rongjie Wei

**Affiliations:** Department of Emergency Management, Sichuan Center for Diseases Control and Prevention, Chengdu, 610041 China

**Keywords:** Bacillary dysentery, Spatial autocorrelation, Spatio-temporal analysis, China

## Abstract

**Background:**

Bacillary dysentery (BD) is a common infectious disease in China and causes enormous economic burdens. The purpose of this study was to describe the epidemiological characteristics of BD and to identify its possible hot spots and potentially high-risk areas in Sichuan province of China.

**Methods:**

In this study, we collected monthly BD incidence reports of 181 counties in Sichuan province, China, from January 2011 to December 2019. Descriptive statistics were used to evaluate the epidemic characteristics of BD. Moran’s I index was applied to investigate the yearly patterns of the spatial distribution. And spatio-temporal scanning statistics with the spatial unit set as county and the temporal unit set as month were used to investigate the possible high-risk region. Meanwhile, the circular moving windows were also employed in the spatio-temporal scanning to scan the study areas.

**Results:**

The annual incidence of BD ranged between 16.13/100,000 and 6.17/100,000 person-years from 2011 to 2019 in Sichuan. The majority of the cases were children aged 5 years or younger. For the descriptive statistics, a peak from May to October was observed in temporal analysis, the epidemics were mainly concentrated in the northwest and southwest of Sichuan in spatial analysis. After 2016, the scope of BD significantly narrowed and severe epidemic areas were relatively stable. For the spatial autocorrelation analysis, a high global autocorrelation was observed at the county level, and the high–high clusters mainly distributed in the northwest and southwest of Sichuan. For the spatio-temporal scanning, the spatiotemporal clusters of BD occurred every year from 2011 to 2019. The most likely cluster areas mainly distributed in the southwest and northwest of Sichuan at the beginning, and then gradually concentrated in the southwest. The secondary cluster mainly concentrated in the northwest and its surrounding areas. Moreover, the 2nd secondary cluster was relatively small and mainly distributed in the central area. No clusters were noted in eastern Sichuan.

**Conclusions:**

Based on our current analysis, BD is still a common challenge in Sichuan, especially for counties in the southwest and northwest in summer and autumn. More disease prevention and control measures should be taken in such higher-risk susceptible areas at a certain time to allocate the public health resources rationally, and finally reduce the spread of BD.

## Background

Bacillary dysentery (BD) is a kind of intestinal bacterial infection caused by different strains of Shigella, which is susceptible to all populations and is the leading cause of diarrhoeal deaths throughout adolescence and adulthood [1]. Ingestion of small amounts of bacteria can cause infection, mainly through the fecal–oral route such as the contaminated water, food or human-to-human contact. BD is a major public health problem in many developing countries, leading to 270 million cases and more than 200,000 deaths every year [2, 3]. Although the incidence rate and mortality rate of China have been decreasing in the past 10 years, there is still a considerable burden of this disease [4, 5]. According to the statistics of China infectious disease detection system, 81,075 cases of bacillary and amoebic dysentery were reported nationwide in 2019.

In recent years, the incidence of BD has ranked sixth among various infectious diseases in Sichuan. Sichuan province is located in the southwest of China with complex terrain and climate system. Due to the great differences in geographical conditions, climate, living environment or habits, the epidemiological characteristics of BD were also of great diversity [6]. Since Shigella outbreaks and epidemics were often caused by water or food pollution, especially in poor personal hygiene and crowded environments [7], taking targeted prevention and control measures in epidemic areas are of great significance to reduce the incidence rate of the disease. Previous studies mainly described the epidemiological characteristics of BD in Sichuan during 2004 and 2014 [8]. However, Sichuan province have changed a lot in terms of society, economy, and environment. Considering these changes occurred in recent years, it was necessary to reassess the epidemiological characteristics of BD in Sichuan.

In the current study, we used descriptive method, spatial autocorrelation analysis, and spatio-temporal scanning statistics to assess the incidence of bacillary dysentery, and to identify possible hot spots and potentially high-risk areas of the disease in Sichuan from 2011 to 2019. We speculated that these may enable us to redefine the characteristics of disease epidemics, so as to promote appropriate allocation of public health resources for better disease control and prevention.

## Methods

### Study area

Sichuan is a southwest province of China with a population of approximately 90 million people (26.03° N–34.19° N and 92.21° E–108.12° E). Covers an area of 486,000 km^2^ and divided into 21 prefectures and 183 counties. The geomorphology of Sichuan is complex and there are significant regional climate differences. Its eastern region climate is characterized by a humid subtropical climate zone and an oceanic climate, the southwest region is a subtropical semi-humid climate zone, and the northwest region is a plateau alpine climate zone.

### Data description

Cases of BD in Sichuan were obtained from the China Information System for Disease Control and Prevention. This data covered the study period (2011–2019), and included clinical cases or laboratory confirmed cases. The diagnostic criteria were based on diagnostic criteria for bacillary dysentery and amoebic dysentery by ministry of health of the PRC [9]. The demographic information of the residents of 181 counties (Two counties were established in 2013, so they were not included in the study) were provided by the Sichuan Statistical Bureau. Geographic space information was acquired from the National Fundamental Geographic Information System of China. All methods were carried out in accordance with relevant guidelines and regulations.

### Spatial autocorrelation analysis

The concept of spatial autocorrelation was put forward by Tobler’s first law of geography: spatial autocorrelation refers to the potential interdependence between observed data of some variables in the same distribution area [10]. As a spatial statistical method, global spatial autocorrelation and local spatial autocorrelation are used to describe the relationship between study areas and measure the degree of aggregation or dispersion [11–13]. Moran’s I index is a tool to measure spatial autocorrelation. The global Moran’s I index is used to measures the overall spatial autocorrelation and spatial distribution of the study areas while the local one can be further used to reflects the local spatial autocorrelation and the specific clustering areas [14]. In this study, we used global spatial autocorrelation and local spatial autocorrelation to explore the spatial correlation of bacterial dysentery in Sichuan.

The value of Moran’s I index range from − 1 to + 1. An I > 0 indicates a positive autocorrelation, and the distribution of cases is aggregated in space. An I < 0 indicates a negative autocorrelation and the closer to − 1, the more scattered the cases are. An I = 0 indicates that the cases are randomly distributed in space [15].

The formula for global Moran’s I is:

$$\mathrm{I}=\frac{n\sum_{i=1}^{n}\sum_{j\ne 1}^{n}{w}_{ij}\left({x}_{i}-\overline{x }\right)\left({x}_{j}-\overline{x }\right)}{\sum_{i=1}^{n}\sum_{j\ne 1}^{n}{w}_{ij}{\left({x}_{i}-\overline{x }\right)}^{2}}$$

where n is the number of areas; ${x}_{i}$ and ${x}_{j}$ are the observed values of areas $i$ and $j$; ${w}_{ij}$ is the element in the spatial weight matrix corresponding to the observation pair $i$,$j$; The value for ${w}_{ij}$ is 1 if province $i$ and province $j$ are adjacent. Otherwise, the value is 0 [16].

Regardless of the existence of global spatial autocorrelation, the local Moran’s I index can be used to find the hot spots and local autocorrelation that may be concealed [17]. The spatial correlation patterns obtained from the local Moran’s I index can be classified into four types, which are shown by the local indicators of spatial autocorrelation (LISA): low–high cluster (LH, which indicated that the low cluster areas were surrounded by high cluster areas), high–low cluster (HL, which indicated that the high cluster areas were surrounded by other low cluster areas), low–low cluster (LL, which indicated the cold spot), and high–high cluster (HH, which indicated the hot spot) [18, 19].

The formula for local Moran’s I is:$\mathrm{I}=\frac{n}{{S}_{0}}\sum_{i}\sum_{j}{w}_{ij}\left({y}_{i}-\overline{y }\right)\left({y}_{j}-\overline{y }\right)/\sum_{i}{\left({y}_{i}-\overline{y }\right)}^{2}$

$${S}_{0}=\sum_{i}\sum_{j}{w}_{ij}$$

where ${y}_{i}$ represents the incidence rate in areas $i$, ${y}_{j}$ represents the incidence rate in areas $j,$ $\overline{y }$ indicates the mean value, ${S}_{0}$ is the sum of ${w}_{ij}$ [20] We used global Moran’s I and local Moran’s I statistic and LISA map to explore the spatial correlation of BD in Sichuan in ArcGIS 10. 6 software.

### Spatio-temporal cluster analysis

We used the spatio-temporal scan statistics which introduced by Kulldorff to detect the center and radius of the aggregation area [21, 22]. The basic principles of spatio-temporal scan is based on a discrete Poisson model [23]. In this approach, the theoretical incidence number of each scanning window is calculated and compared with the actual incidence number to construct the log likelihood ratio (LLR) for statistical inference, and use Monte Carlo randomization method to evaluate statistical significance to explore the largest possible gathering area [24].

The formula for LLR is: $\mathrm{LLR}=\mathrm{log}\left\{{\left(c/\mu \right)}^{C}{\left[\left(C-c\right)/\left(C-\mu \right)\right]}^{\left(C-c\right)}\right\}$ Where $C$ represents the total number of cases, $c$ denotes the actual number of cases, and $\mu$ represents the expected number of cases. For each possible spatio-temporal aggregation area, when P < 0.05, as the LLR increased, the possibility that regarded the area covered by the scanning dynamic window as the cluster increased [25]. We chose the window area with the largest LLR value as the most likely aggregation area, which represents this high-risk region [26]. And other statistically significant Windows were secondary and tertiary probable aggregation areas in turn. In this study, SatScan 9.11 software was used for spatial–temporal statistical, and Arc GIS 10.6 software was used for visual presentation of the scanning results. In spatio-temporal scan analysis, the spatial unit was set as county (a total of 181 counties in Sichuan province); the temporal unit was set as month (a total of 108 months from 2011 to 2019). Circular moving windows were set to scan the study area. Radiuses of circles were set to vary continuously from zero to 50% of the population at risk, and the time size was set as 50% of the total study period. The number of Monte Carlo randomization was set to 999, and the time frame for scanning analysis was set to 1 month.

## Results

### Demographic characteristics

The incidence rates of BD varied by age, gender and population classification. From 2011 to 2019, the annual incidence ranged between 16.13 and 6.17 per 100,000 person-years in Sichuan. Table 1 showed the detailed demographic characteristics of BD cases. The highest incidence rate was noted in children aged less than 1 year old (incidence rates, 84.94–207.49 per 100,000 person-years), and the lowest was noted in cases aged between 35 and 40 years old (incidence rates, 2.13–7.85 per 100,000 person-years). The male-to-female ratio showed a relatively declining trend, ranging from 1.21:1 in 2011 to 1.05:1 in 2019). Simultaneously, we found that most of the BD cases were scattered children or farmer (Table 1).

**Table 1:** Incidence rates (cases/100,000 person-years) of BD in Sichuan province, 2011–2019

	2011	2012	2013	2014	2015	2016	2017	2018	2019
Annual incidence	16.13	12.96	11.60	8.76	7.31	6.85	6.65	5.79	6.17
*Age (incidence)*									
< 1	207.49	223.83	230.61	167.37	149.39	121.55	122.46	111.32	84.94
1~	134.99	98.52	96.42	78.15	63.50	63.45	59.85	59.33	47.00
2~	73.71	52.32	45.19	43.01	34.78	35.83	31.49	30.27	28.57
3~	52.08	38.01	39.72	28.90	24.60	25.68	22.58	15.69	18.47
4~	33.84	30.90	28.43	20.34	17.41	16.70	19.97	13.63	17.54
5~	27.97	24.43	23.88	20.27	14.19	16.76	13.98	15.50	12.09
6~	21.88	19.37	22.52	12.22	13.89	13.12	12.57	10.62	11.52
7~	17.18	11.76	10.84	7.19	7.47	5.67	4.93	4.60	9.28
8~	13.97	8.81	10.09	5.39	4.46	3.64	3.48	3.75	7.77
9~	13.95	13.38	14.86	8.74	8.19	7.61	6.17	7.06	6.04
10~	10.58	7.32	6.98	4.81	4.15	4.05	4.09	3.70	4.11
15~	6.74	6.04	5.17	3.78	3.01	3.95	3.20	2.80	4.21
20~	9.49	6.94	5.86	3.92	3.32	3.26	3.49	2.80	5.54
25~	13.94	10.31	8.47	6.33	5.37	4.79	5.41	4.46	3.73
30~	9.83	11.00	8.98	6.21	5.60	4.91	5.08	4.33	4.46
35~	7.85	6.44	5.04	3.86	2.59	2.13	2.58	1.90	2.13
40~	8.99	7.35	6.08	4.41	3.11	2.89	2.84	2.49	2.72
45~	13.18	7.29	5.22	3.91	3.34	3.05	3.03	2.56	2.72
50~	7.39	8.87	8.12	6.65	5.69	6.51	7.19	5.76	3.89
55~	10.88	8.45	6.64	4.69	3.32	2.74	2.48	2.14	3.59
60~	15.11	11.05	8.31	7.17	5.57	5.33	4.56	3.78	3.61
65~	13.65	12.15	10.18	8.11	6.37	5.56	4.89	4.10	4.30
70~	16.58	13.24	13.39	8.82	8.16	6.34	6.70	4.43	4.61
75~	14.86	13.61	13.46	8.66	8.12	7.37	6.75	6.39	8.28
80~	19.82	13.45	10.67	8.95	6.99	5.20	4.87	4.72	5.91
≥ 85	35.50	10.41	7.59	9.50	7.67	6.33	7.34	5.02	5.50
*Sex (incidence)*									
Male	17.40	13.47	11.53	8.89	7.57	7.14	6.63	5.80	6.27
Female	14.82	12.43	11.69	8.64	7.06	6.56	6.67	5.78	6.07
Male-to-female ratio	1.21	1.12	1.02	1.05	1.05	1.10	1.01	1.02	1.05
*Population classification (%)*									
Scattered children	34.68	35.27	38.97	40.08	41.31	39.87	39.32	40.35	33.99
School-children	8.97	8.91	9.68	8.66	9.07	9.98	9.30	9.92	12.98
Nursery children	3.27	2.89	3.12	3.25	3.16	4.45	4.02	4.70	5.50
Farmer	32.25	33.40	30.89	30.63	29.75	28.86	31.00	28.60	30.73
others	20.83	19.54	17.35	17.38	16.71	16.85	16.36	16.43	16.80

### Temporal characteristics

**Fig. 1:**
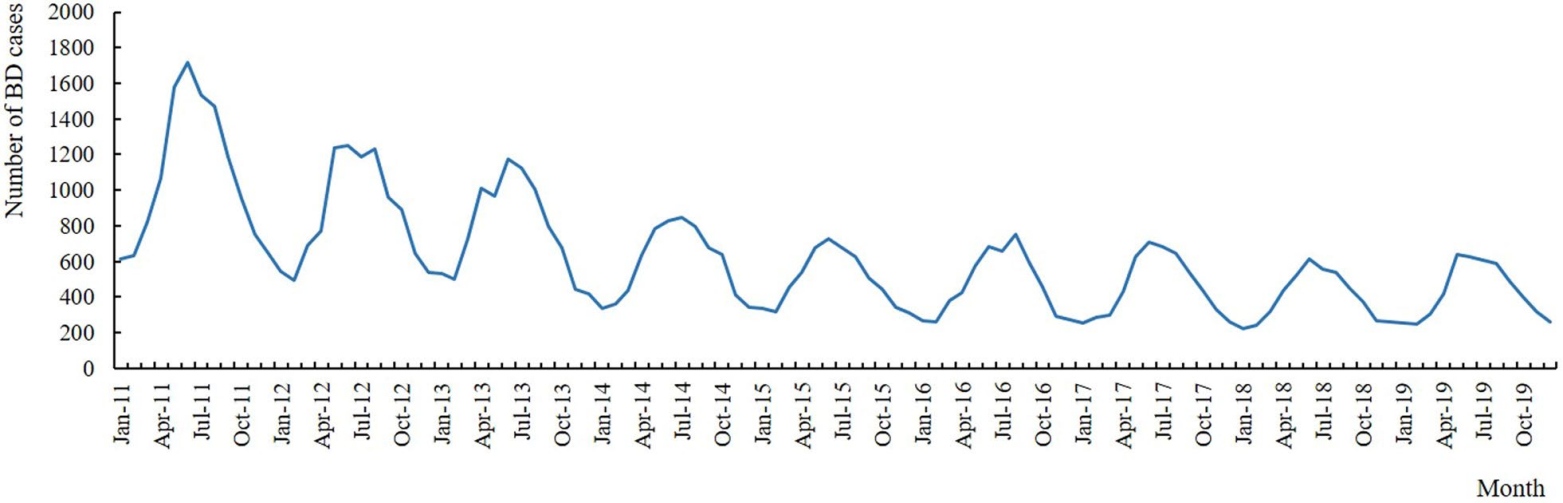
Monthly distribution of BD cases in Sichuan province, 2011–2019

The monthly distribution of BD cases in Sichuan was shown in Fig. 1, which presented clear seasonal peak with the wave-like degressive tendency. Obviously, the incidence peak appeared between May and October, which accounted for 64.18% of all reported cases. The fewest cases were reported between January and February, accounting for 10.02% of all reported cases.

### Spatial characteristics

**Fig. 2:**
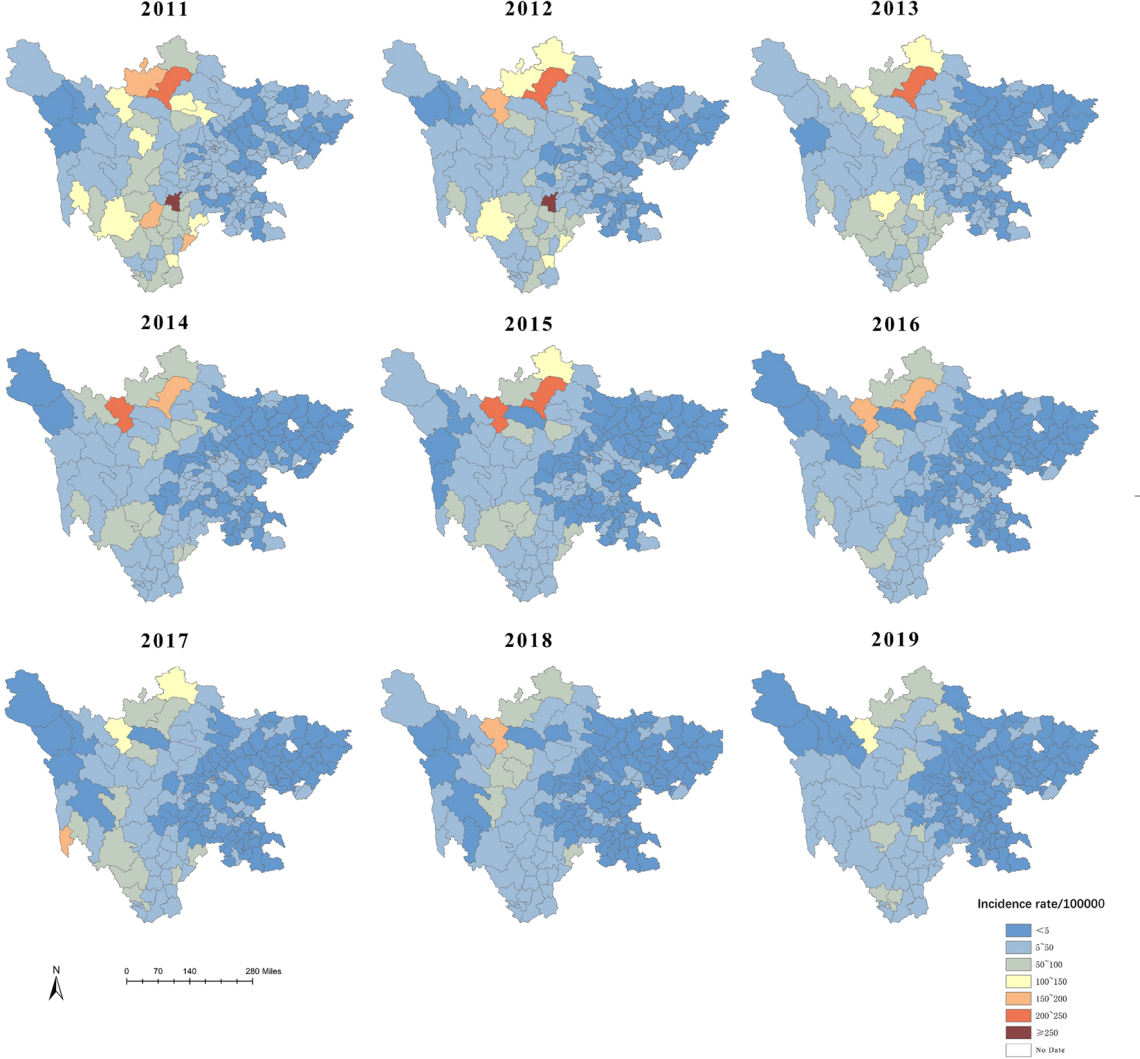
The incidence of BD at the county level in Sichuan province, 2011–2019

During 2011–2019, the incidence of BD reported by all counties varied greatly and the distribution was heterogeneous. Figure 2 showed the yearly incidence rates of BD at the county level in Sichuan, which indicated that the incidence was relatively high from 2011 to 2013, and the epidemics were mainly concentrated in the northwest and southwest. After 2016, the scope of BD significantly narrowed and severe epidemic areas were relatively stable. As for 2019, 112 counties (61.88%) reported incidence rates less than 5 per 100,000 person-years. In general, the incidence rates of BD in most areas of Sichuan have been decreasing year by year, especially in the southwest.

### Spatial autocorrelation analysis

**Fig. 3:**
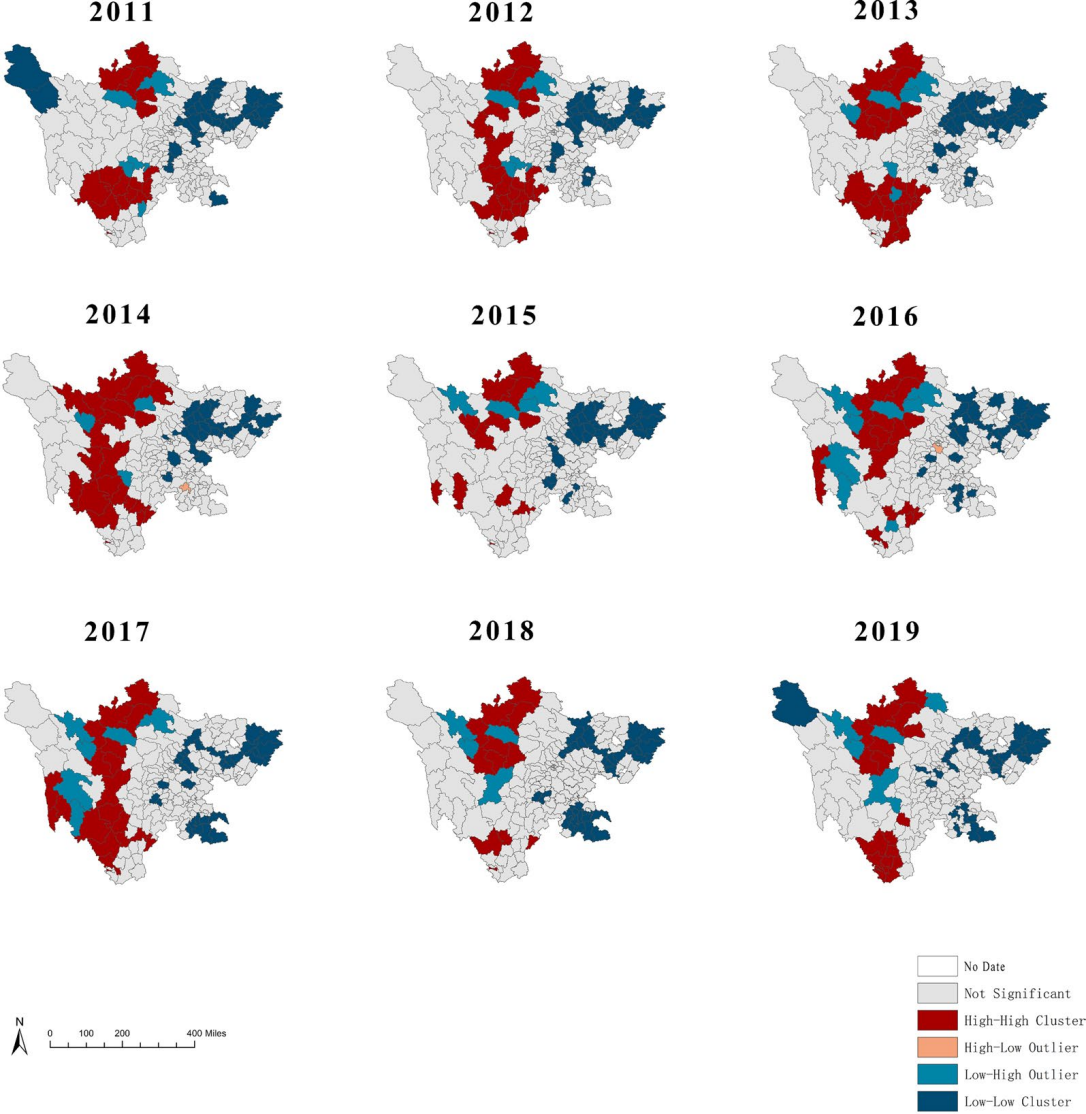
LISA maps for the incidence of BD in Sichuan province, 2011–2019

The global spatial autocorrelation analysis of BD found that the annual global Moran’s I values ranged from 0.369 to 0.405, which suggested a statistically high level of clustering (*p* < 0.01). The results showed that the distribution of BD in Sichuan was not random, moreover, a high global autocorrelation was noted at the county level (Table 2). Local autocorrelation analysis results were shown in Fig. 3. The LISA map showed that the high–high clusters were mainly distributed in Aba prefecture and Liangshan prefecture in the northwest and southwest of Sichuan, including Rangtang, Hongyuan, Jinchuan, Xichang, Yanyuan. While the low–low clusters were mainly distributed in eastern districts including Wanyuan, Xuanhan, Dachuan. Differing from other counties in the northwest, have shown a Low–high cluster were detected in Luhuo and Sertar in Ganzi Prefecture in recent years.

**Table 2:** Results of the spatial autocorrelation test on BD cases in Sichuan province, 2011–2019

Year	Moran’s I	Z score	P value
2011	0.368921	8.704542	< 0.0001
2012	0.327338	7.875358	< 0.0001
2013	0.472139	10.957065	< 0.0001
2014	0.436069	10.329668	< 0.0001
2015	0.341136	8.384805	< 0.0001
2016	0.395031	9.434870	< 0.0001
2017	0.393783	9.137914	< 0.0001
2018	0.428190	10.340111	< 0.0001
2019	0.404887	9.333226	< 0.0001

### Spatio-temporal cluster analysis

**Fig. 4:**
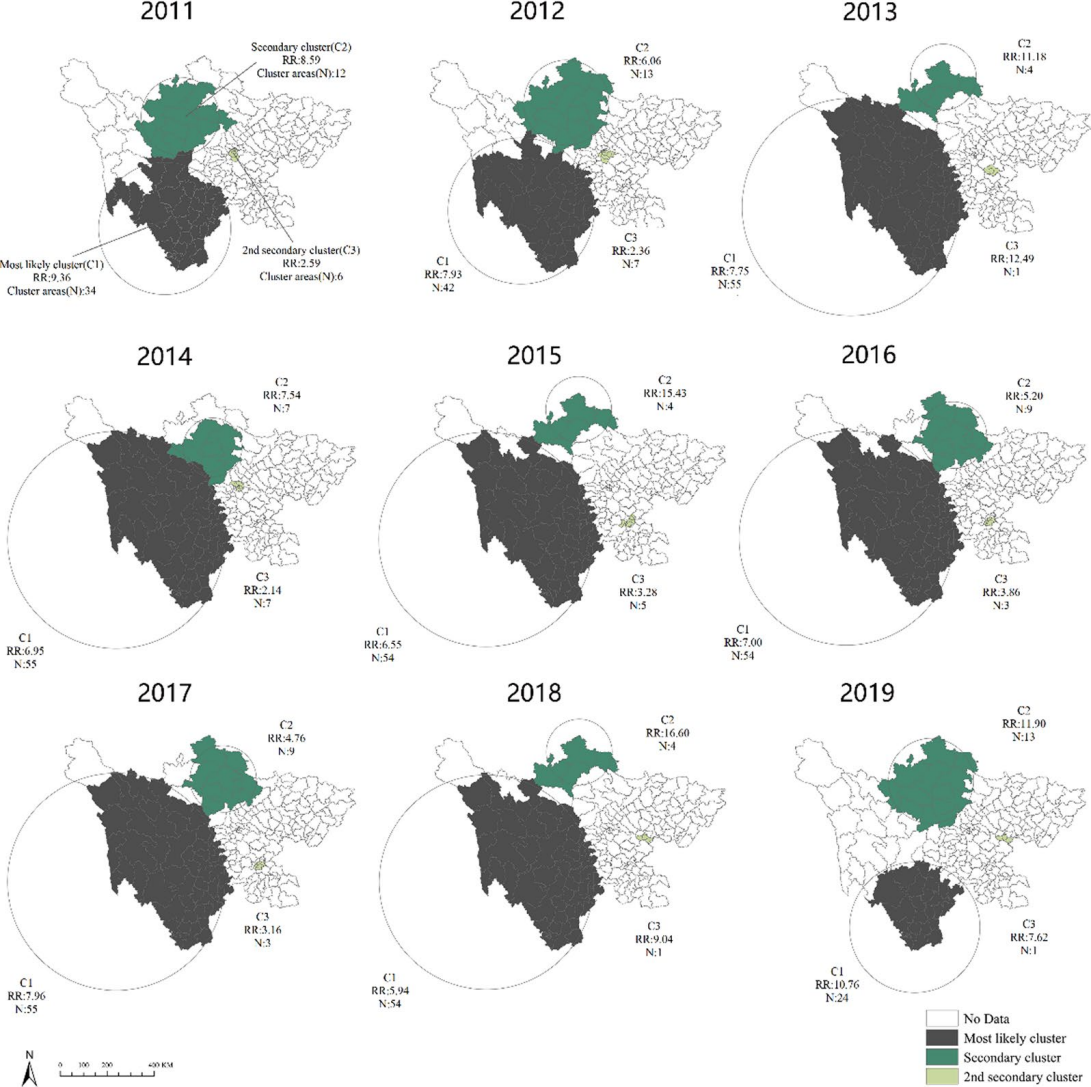
Spatio-temporal clusters of BD in Sichuan province, 2011–2019. Parameters N and RR indicate cluster areas number and relative risk of each cluster, respectively

Spatiotemporal clusters of BD cases appeared annually during the study periods. The most majority of the clusters occurred in May–October, which was the same as the major peak of incidence of BD in Sichuan. The most likely cluster included 34 counties in 2011, of which the cluster center was (27.69 N, 101.38 E) and the cluster radius was 253.85 km. The cluster time was from April to September in 2011, with a relative risk (RR) value of 9.36 (P < 0.0001). Similarly, most likely clusters were also observed in the other years (Table 3 and Fig. 4). Twenty-four counties were always included in the most likely clusters during 2011–2019, most of which were located in Liangshan (70.83%) and Panzhihua prefecture (20.83%). The secondary cluster centers were always located in Aba prefecture in Northwest Sichuan. It was worth noting that only Hongyuan county was included in the secondary likely clusters each year from 2011 to 2019.The 2nd secondary cluster were relatively smaller and distributed in the central region of Sichuan. The cluster centers were located in Chengdu in 2011, 2012 and 2014, and moved to Zigong from 2015 to 2017, finally concentrated in Suining in 2018 and 2019.

**Table 3:** Spatio-temporal cluster results on BD incidence at county level, 2011–2019

Year	Cluster	Cluster areas	Cluster center/radius (km)	Cluster time	RR	LLR	P value
2011	Most likely cluster	34	(27.69 N, 101.38 E)/253.85	2011/4/1 to 2011/9/30	9.36	4307.35	< 0.0001
	Secondary cluster	12	(32.00 N, 101.97 E)/149.81	2011/4/1 to 2011/9/30	8.59	548.74	< 0.0001
	2nd secondary cluster	6	(30.59 N, 104.02 E)/16.86	2011/4/1 to 2011/9/30	2.59	246.70	< 0.0001
2012	Most likely cluster	42	(28.42 N, 100.85 E)/280.99	2012/4/1 to 2012/9/30	7.93	3133.91	< 0.0001
	Secondary cluster	13	(32.58 N, 102.59 E)/163.64	2012/3/1 to 2012/8/31	6.06	288.83	< 0.0001
	2nd secondary cluster	7	(30.61 N, 104.11 E)/20.15	2012/4/1 to 2012/9/30	2.36	172.56	< 0.0001
2013	Most likely cluster	55	(28.68 N, 99.34 E)/417.29	2013/3/1 to 2013/8/31	7.75	3003.06	< 0.0001
	Secondary cluster	4	(33.68 N, 102.90 E)/126.37	2013/7/1 to 2013/12/31	11.18	227.52	< 0.0001
	2nd secondary cluster	1	(30.07 N, 104.74 E)/0.00	2013/4/1 to 2013/4/30	12.49	182.34	< 0.0001
2014	Most likely cluster	55	(28.68 N, 99.34 E)/417.29	2014/4/1 to 2014/9/30	6.95	1952.17	< 0.0001
	Secondary cluster	7	(32.24 N, 103.04 E)/113.85	2014/6/1 to 2014/11/30	7.54	163.73	< 0.0001
	2nd secondary cluster	7	(30.73 N, 104.04 E)/19.23	2014/4/1 to 2014/9/30	2.14	87.08	< 0.0001
2015	Most likely cluster	54	(28.68 N, 99.34 E)/412.13	2015/4/1 to 2015/9/30	6.55	1557.76	< 0.0001
	Secondary cluster	4	(33.68 N, 102.90 E)/126.37	2015/7/1 to 2015/12/31	15.43	275.54	< 0.0001
	2nd secondary cluster	5	(29.39 N, 104.85 E)/20.85	2015/6/1 to 2015/10/31	3.28	87.18	< 0.0001
2016	Most likely cluster	54	(28.68 N, 99.34 E)/412.13	2016/4/1 to 2016/9/30	7.00	1620.57	< 0.0001
	Secondary cluster	9	(32.63 N, 103.45 E)/127.80	2016/4/1 to 2016/9/30	5.20	126.32	< 0.0001
	2nd secondary cluster	3	(29.39 N, 104.85 E)/18.29	2016/4/1 to 2016/9/30	3.86	84.39	< 0.0001
2017	Most likely cluster	55	(28.68 N, 99.34 E)/417.29	2017/4/1 to 2017/9/30	7.96	1884.31	< 0.0001
	Secondary cluster	9	(32.63 N, 103.45 E)/127.80	2017/5/1 to 2017/10/31	4.76	103.72	< 0.0001
	2nd secondary cluster	3	(29.39 N, 104.85 E)/18.29	2017/5/1 to 2017/10/31	3.16	59.54	< 0.0001
2018	Most likely cluster	54	(28.68 N, 99.34 E)/412.13	2018/3/1 to 2018/8/31	5.94	1086.65	< 0.0001
	Secondary cluster	4	(33.68 N, 102.90 E)/126.37	2018/6/1 to 2018/11/30	16.60	243.28	< 0.0001
	2nd secondary cluster	1	(30.41 N, 105.42 E)/0.00	2018/5/1 to 2018/10/31	9.04	207.74	< 0.0001
2019	Most likely cluster	24	(26.95 N, 101.97 E)/250.58	2019/5/1 to 2019/10/31	10.76	1963.02	< 0.0001
	Secondary cluster	13	(32.58 N, 102.59 E)/163.64	2019/4/1 to 2019/9/30	11.90	485.82	< 0.0001
	2nd secondary cluster	1	(30.41 N, 105.42 E)/0.00	2019/5/1 to 2019/10/31	7.62	165.76	< 0.0001

## Discussion

In the current study, we investigated the epidemiological and spatiotemporal characteristics of BD for the purpose of a good understanding of the disease’s distribution in Sichuan. Public health researchers are usually interested in using data visualization methods to describe the distribution of diseases. The reason is that the visualization of high-risk disease areas can guide managers to prioritize the optimal allocation of investment, personnel, and services, so as to realize the optimal allocation of resources among regions [27]. In this study, the incidence of BD at the county level in Sichuan from 2011 to 2019 was used to discuss the epidemiological characteristics of the disease and investigate its spatial and temporal distribution rules and possible hot spots.

The incidence of BD in Sichuan showed a downward trend from 2011 to 2019. One possible explanation was that rapid economic growth has resulted in significant improvements in water supply and sanitation facilities, as well as significant changes in population hygiene practices [28]. In terms of age, BD mainly affected children under the age of 5, followed by children between the ages of 5 and 9, which was consistent with the prior studies [29–31]. We speculated that the poor awareness of disease prevention and poor hygiene could lead to the infections, moreover, hypo immunity may further cause the disease progression. Under this situation, targeted prevention and control measures for children could have great public health significance to control the spread of BD, which has already been confirmed by prior study [32], Our study found that the occupations of the cases were mainly scattered children and farmers, we hypothesis that this may be related to the low prevalence of running water and sanitary toilets in rural areas.

For the temporal characteristics, there were new cases every month, and the incidence rates showed an obvious seasonal distribution. The same as the prior studies [33–35], the peak of BD appeared early and lasted from May to October. As we all know, the occurrence of intestinal infectious diseases is related to climatic factors such as sunshine, temperature, humidity, and the quality of food or drinking water [36]. The high temperature and humidity of Sichuan in summer and autumn accelerate bacterial reproduction. Once the food and water were contaminated, it was easy to get BD.

In the current study, the distribution of BD was heterogeneous. The areas of high incidence mainly concentrated in the northwest and southwest regions where the economy was relatively backward. On the contrary, the incidence was low in the eastern and central regions with a relatively developed economy, which suggested that the more developed the regional economy was, the lower the incidence rates were. And the prior studies have also indicated that economic development always means superior water supply, more complete sanitation facilities and better medical care, which could to some extent prevent the further spread of BD and reduce its incidence rates [37–39]. It also explained why farmers were more easily infected by BD. Besides, the animal husbandry, a factor that promote the spread of bacteria, was more common in western Sichuan, which may also play a role in the epidemic of BD [8].

According to the spatial autocorrelation analysis, we found that BD was not randomly distributed at county level in Sichuan from 2011 to 2019. According to the LISA map, high–high clusters were mainly distributed in in the northwest and southwest of Sichuan. The hot spots of geographical environment presented similar characteristics, including relief, sparsely populated and relatively backward economy. But a low–high cluster was detected in Ganzi Prefecture, which also belongs to the western areas. We speculated that the effective infectious disease control measures taken in Ganzi Prefecture in recent years have improved the epidemic. However, inaccurate diagnosis or delayed reports of BD could also cause the difference detected in our study. Besides, Ganzi Prefecture had a unique plateau climate, which has been reported to be not suitable for the reproduction of the bacteria for its coldness and dryness [40].

For the results of the spatiotemporal cluster analysis of BD in Sichuan, we noted that the most likely clusters located in the southwest and northwest of Sichuan, and gradually concentrated in the southwest, which partially indicated that the disease control measures taken these years have made sense, especially in the northwest. Simultaneously, we found that the 2nd secondary clusters mainly concentrated in Chengdu and its surrounding areas, which may be due to the high population density and high population mobility in such areas. In the current analysis, it was worth noting that there were no clusters existed in eastern areas, where the terrain was relatively flat and the altitude was relatively low. Prior study has confirmed that developed economy, high altitude, relief and minority areas were the risk factors for BD [41], which may explain the phenomenon found in eastern areas to some extent.

Limitations should be considered when interpreting the findings of this study. First, the data of this study came from the China’s current surveillance system, which may have missed some BD cases for several reasons as unreported, undiagnosed or misdiagnosed. Second, this study was confined to Sichuan province, a boarder range of study was needed in our following studies.

## Conclusion

BD is still a common challenge in Sichuan, especially for counties in the southwest and northwest from May to October. In such higher-risk susceptible areas, targeted measures should be taken at a certain time to reduce the spread of BD. Further researches should focus more on the influencing factors, such as the environmental and socio-economic factors, to achieve better understanding of the disease.

## Data Availability

The datasets generated and analyzed during the current study are not publicly available due to the fact that it contains personal information, but are available from the corresponding author on reasonable request.

## Abbreviations

BD: Bacillary dysentery
LISA: Local indicators of spatial autocorrelation
LL: Low–low cluster
LH: Low–high cluster
HL: High–low cluster
HH: High–high cluster
LLR: Log likelihood ratio

## References

[1] Fried M (2007). Acute gastrointestinal infections. Gastroenterologe.

[2] Yum LK, Byndloss MX, Feldman SH, Agaisse H (2019). Critical role of bacterial dissemination in an infant rabbit model of bacillary dysentery. Nat Commun.

[3] Khalil IA, Troeger C, Blacker BF, Rao PC, Brown A, Atherly DE, Brewer TG, Engmann CM, Houpt ER, Kang G (2018). Morbidity and mortality due to Shigella and enterotoxigenic *Escherichia coli* diarrhoea: the Global Burden of Disease Study 1990–2016. Lancet Infect Dis.

[4] Zhang Z, Lai S, Yu J, Geng Q, Yang W, Yu C, Wu J, Jing H, Yang W, Li Z (2017). Etiology of acute diarrhea in the elderly in China: a six-year observational study. PLoS ONE.

[5] Kotloff KL, Nataro JP, Blackwelder WC, Nasrin D, Farag TH (2013). Burden and aetiology of diarrhoeal disease in infants and young children in developing countries (the Global Enteric Multicenter Study, GEMS): a prospective, case–control study. Lancet London.

[6] Meng Q, Liu X, Xie J, Xiao D, Wang Y, Deng D (2019). Epidemiological characteristics of bacillary dysentery from 2009 to 2016 and its incidence prediction model based on meteorological factors. Environ Health Prev Med.

[7] Taneja N, Mewara A (2016). Shigellosis: epidemiology in India. Indian J Med Res.

[8] Ma Y, Zhang T, Liu L, Lv Q, Yin F (2015). Spatio-temporal pattern and socio-economic factors of bacillary dysentery at county level in Sichuan Province, China. Sci Rep.

[9] Ministry of health of the PRC: Diagnostic criteria for bacillary dysentery and amoebic dysentery. 2008; WS 287-2008.

[10] Tobler WR (1970). A computer movie simulating urban growth in the Detroit region. Econ Geogr.

[11] Flahaut B, Mouchart M, Martin ES, Thomas I (2003). The local spatial autocorrelation and the kernel method for identifying black zones: a comparative approach. Accid Anal Prev.

[12] Mattsson BJ, Zipkin EF, Gardner B, Blank PJ, Sauer JR, Royle JA (2013). Explaining local-scale species distributions: relative contributions of spatial autocorrelation and landscape heterogeneity for an avian assemblage. PLoS ONE.

[13] Viladomat J, Mazumder R, Mcinturff A, McCauley DJ, Hastie T (2014). Assessing the significance of global and local correlations under spatial autocorrelation: a nonparametric approach. Biometrics.

[14] Tillé Y, Dickson MM, Espa G, Giuliani D (2018). Measuring the spatial balance of a sample: a new measure based on the Moran’s I index. Spat Stat.

[15] Mao Y, Zhang N, Zhu B, Liu J, He R (2019). A descriptive analysis of the spatio-temporal distribution of intestinal infectious diseases in China. BMC Infect Dis.

[16] Wu X, Hu S, Kwaku AB, Li Q, Luo K, Zhou Y, Tan H (2017). Spatio-temporal clustering analysis and its determinants of hand, foot and mouth disease in Hunan, China, 2009–2015. BMC Infect Dis.

[17] Zhu B, Fu Y, Liu J, Ying M (2017). Notifiable sexually transmitted infections in China: epidemiologic trends and spatial changing patterns. Sustainability.

[18] Waldhör T (2010). The spatial autocorrelation coefficient Moran’s I under heteroscedasticity. Stat Med.

[19] Anselin L (2010). Local indicators of spatial association—LISA. Geogr Anal.

[20] Anselin L (1995). Local Indicator of Spatial Association—LISA. Geogr Anal.

[21] Kulldorff M (1997). A spatial scan statistic. Commun Stat Theory Methods.

[22] Kulldorff M, Heffernan R, Hartman J, Assunçao R, Mostashari F (2005). A space–time permutation scan statistic for disease outbreak detection. PLoS Med.

[23] Kulldorff M, Huang L, Pickle L, Duczmal L (2006). An elliptic spatial scan statistic. Stat Med.

[24] Kleinman KP, Abrams AM, Kulldorff M, Platt R (2005). A model-adjusted space–time scan statistic with an application to syndromic surveillance. Epidemiol Infect.

[25] Zhu B, Liu J, Fu Y, Zhang B, Mao Y (2018). Spatio-temporal epidemiology of viral hepatitis in China (2003–2015): implications for prevention and control policies. Int J Environ Res Public Health.

[26] Kulldorff M, Feuer EJ, Miller BA, Freedman LS (1997). Breast cancer clusters in the northeast United States: a geographic analysis. Am J Epidemiol.

[27] Abolfazl M, Abbas A, Mostafa K (2014). Spatial and spatio-temporal analysis of human brucellosis in Iran. Trans R Soc Trop Med Hyg.

[28] He-Yan WU, Xiao WH, Guang-Zhi LU (2018). Rural environmental sanitation in Guangdong, 2011–2016. Mod Prev Med..

[29] Wei XY, Tian KC, You LU (2012). Epidemic characteristics and etiological analysis of bacillary dysentery in Guizhou province during the period of 2007–2010. Pract Prev Med..

[30] Wang X, Zhang Y, Xing DG, Wen T, Meng QY, Tang L (2018). Analysis on the epidemiological characteristics and temporal-spatial clusters of bacillary dysentery incidence in Chongqing from 2005 to 2015. Chin J Dis Control Prev..

[31] Liu D, Qiu L, Shi Y (2019). Epidemiological analysis of spatio-temporal distribution of bacterial dysentery in Shaanxi Province, 2004–2017. Chin J Health Stat..

[32] Gao L (2017). Current status of research on bacterial dysentery. Occup Health..

[33] Cui CY, Wei Z, Office C (2019). Epidemiological characteristics of bacillary dysentery in Xi’an City from 2012–2017. Occup Health..

[34] Xu C, Xiao G, Wang J, Zhang X, Liang J (2018). Spatiotemporal risk of bacillary dysentery and sensitivity to meteorological factors in Hunan Province, China. Int J Environ Res Public Health.

[35] Chen Y, Liu X, Kong Q, Huang Y (2019). Epidemiological characteristics of bacillary dysentery in Xicheng District of Beijing from 2014 to 2017. J Med Pest Control..

[36] Dongyu XU, Liu B, Zheng L, Lou Y, Department C. Hierarchical clustering analysis on the incidence of five types of common intestinal infectious diseases in China. J Prev Med Inf. 2018;34(01):9-12.

[37] Von Seidlein L, Kim DR, Ali M, Lee H, Wang XY, Thiem VD, Canh DG, Chaicumpa W, Agtini MD, Hossain A (2006). A multicentre study of Shigella diarrhoea in six Asian countries: disease burden, clinical manifestations, and microbiology. PLoS Med.

[38] Chang ZR, Sun QZ, Pei YX, Zhang J, Sun JL (2014). Surveillance for bacillary dysentery in China, 2012. Dis Surveill.

[39] Wang XY, Tao F, Xiao D, Lee H, Deen J, Gong J, Zhao Y, Zhou W, Li W, Shen B (2006). Trend and disease burden of bacillary dysentery in China (1991–2000). Bull World Health Organ.

[40] Wang X, Zhang Y, Shi Q (2018). Spatial distribution of hot spots of bacterial dysentery and related environmental factors in southwestern China. Dis Surveill.

[41] Wang X, Zhang Y, Ma J (2019). Factors influencing the incidence of bacterial dysentery in parts of southwest China, using data from the geodetector. Chin J Epidemiol.

